# Spontaneous regression of equine sarcoids is an exceptional event

**DOI:** 10.1002/evj.70158

**Published:** 2026-03-11

**Authors:** Sabine Brandt

**Affiliations:** ^1^ Research Group Oncology, Centre of Equine Health and Research Veterinary University Vienna Austria

**Keywords:** bovine papillomavirus, equid species, horse, immune response, sarcoid, spontaneous regression

## Abstract

Sarcoids are benign, yet locally aggressive skin tumours that commonly affect horses and other equid species. The lesions are induced by bovine papillomavirus types 1, 2, and probably 13 in conjunction with other factors including trauma and a genetic predisposition. Although sarcoids have a substantial impact on the health and welfare of affected equids, information on the immune response to bovine papillomavirus infection and resulting sarcoids is limited. However, there is evidence that sarcoid disease is associated with an impaired immune response to bovine papillomavirus infection. This observation agrees with the common perception of sarcoids as persistent lesions. Contrary to this general view, there are reports on the spontaneous regression of skin tumours diagnosed as sarcoids, giving rise to the question whether sarcoids can self‐resolve, and if so, under which circumstances. This review addresses this unresolved issue.

## 1 BOVINE PAPILLOMAVIRUS‐INDUCED SARCOIDS IN EQUID SPECIES

Papillomaviruses (PVs) are a family of small, genetically heterogenous DNA viruses that can induce benign or malignant tumours in vertebrates. As a common feature, all PVs consist of a non‐enveloped capsid harbouring a circular, double‐stranded DNA genome. The capsid is composed of 72 major L1 capsid protein pentamers also known as “capsomers” that form an icosahedral particle. A minimum of 12 minor L2 protein monomers embedded between the capsomers complete the capsid. The viral genome contains open reading frames (ORF) for early (E) regulatory (notably E1, E2 and E4) and up to three transforming proteins (E5, E6 and E7), and for the late (L) capsid proteins L1 and L2. A non‐coding long control region (LCR) located downstream of the L1 ORF provides elements that are essential for viral replication and transcription.^1^

Papillomaviruses are usually host‐specific and have a pronounced tropism for cutaneous and mucosal keratinocytes. Their productive life cycle is tightly linked to the differentiation program of these cells. Following virion entry into basal keratinocytes and disassembly, the virus expresses its early regulatory and transforming proteins in the basal and suprabasal epidermal layers. In the differentiated cells of the spinous and granular layers, the viral genome replicates by one to two logs. The capsid proteins are produced in the granular and terminal squamous layers where the viral genomes are packaged by directed assembly of L1 and L2. Resulting new virions are subsequently shed through desquamation.^2^ PV infection is not always productive. The viral genome can be maintained in infected cells as extrachromosomal elements termed episomes^3^ or can integrate into the host cell genome.^4^

The genus delta‐(δ‐)PVs differs substantially from other PV genera. It groups a small number of PVs that also or preferentially infect dermal fibroblasts and—perhaps for this reason—have a wider host range. The feature is best documented for bovine δ‐PV types 1 and 2 (BPV1, BPV2), which also infect horses and other equid species such as donkeys, hinnies, mules, and zebras.^5^ BPV1 and BPV2 share a genetic identity of >90% and antisera induced by BPV1 or BPV2 L1 virus‐like particles (VLPs) can robustly (cross‐) neutralise the heterologous and the homologous BPV type in vitro and in vivo.^6^, ^7^ In bovids, BPV1 and BPV2 infect the epidermis and the underlying dermis and induce fibropapillomas (cow warts) that usually regress spontaneously after several months.^3^, ^8^ In equids however, BPV1, BPV2, and possibly the bovine δ‐PV types 13 and 14 (BPV13, BPV14) are the causative agents of benign, yet locally aggressive skin tumours termed sarcoids.^5^, ^9^, ^10^ In Central Europe and the Eastern USA, sarcoids are predominantly associated with BPV1 infection, whilst sarcoids in Southern Europe, the Western USA or Western Canada are commonly induced by BPV2.^10^, ^11^, ^12^ BPV13 has been first detected from equine sarcoids in Brazil.^9^ A possible association of BPV14 with sarcoid disease is under investigation.^10^

Sarcoids constitute the most common tumour type in horses and other equid species. Depending on the data source, up to 67% of all neoplasms and 70% of all skin lesions in equids are diagnosed as sarcoids.^13^, ^14^ A disease prevalence of 5.8%, 15.2%, and 12% has been reported for horses^15^ and donkeys in the UK,^16^ and for Franches‐Montagnes horses and Swiss Warmbloods in Switzerland.^17^, ^18^ At the Veterinary University Vienna, Austria, the sarcoid case load is 1.5%.^19^ At the Faculty of Veterinary Medicine in Ghent, Belgium, 2.2% of admitted equids are sarcoid‐affected.^13^ Similar data are reported for Veterinary Hospitals in the USA and Canada.^20^, ^21^ Given that uncomplicated lesions are usually treated by local practitioners and that some sarcoid‐bearing equids are left untreated, the sarcoid prevalence of 5.8% reported for the UK likely approximates the worldwide occurrence of sarcoid disease.

Sarcoids mainly develop in young animals and often compromise their use as leisure, sport, breeding or working equids because of their location and/or severity. As a consequence, the disease can drastically reduce or even annul the animals' (re‐) sale value.^22^ Response of sarcoids to current standard of care such as surgical excision, cryo‐, and chemotherapy or combinations of these remains unsatisfactory. In a substantial number of cases, treatment leads to disease recurrence in a more aggressive, multiple form due to its viral aetiology.^22^, ^23^, ^24^, ^25^, ^26^ Consequently, sarcoids still constitute the number‐one dermatologic reason for euthanasia although the lesions do not metastasise.^14^, ^23^

Sarcoids present in various clinical forms that may require different therapeutic approaches. Therefore, sarcoids are classified according to their clinical manifestations into six types: occult, verrucous, nodular, fibroblastic, mixed, or malevolent sarcoids that invade the lymphatics.^27^ Occult sarcoids are round to oval lesions that are alopaecic or display fewer, shorter and/or depigmented hair. The skin is slightly thickened and mildly hyperkeratotic, and subepidermal miliary nodules or plaques are detectable by careful palpation.^27^, ^28^ Occult sarcoids can be easily confounded with fungal or bacterial infections, alopaecia areata, skin rubs, abrasions, or scratches.^27^, ^28^, ^29^ Verrucous sarcoids are hairless, hyperkeratotic lesions of warty appearance that may occasionally display areas of ulceration. Extensive flaking of the epidermal surface is a typical feature. Whilst the clinical diagnosis of extensive verrucous sarcoids is straightforward, small single lesions can be confounded inter alia with dermatophytosis, dermatophilosis, EcPV‐induced transient papillomatosis, irritation‐induced hyperkeratosis, superficial recurrent abrasions or equine sarcoidosis.^28^, ^29^ Nodular sarcoids present as firm, pedunculated or sessile subcutaneous nodules that can vary in size. They are covered by apparently intact skin.^27^ Fibroblastic sarcoids are characterised by their fleshy, appearance that is caused by complete destruction of the epidermis. They present with pronounced ulceration and serum exudation and are commonly infected by bacteria. Some fibroblastic sarcoids closely resemble proud flesh, that is, exuberant granulation tissue, especially at sites of previous injury.^27^ Mixed sarcoids are conglomerates of different sarcoid types in various combinations with high progressive potential.^27^ Malevolent sarcoids constitute the most aggressive sarcoid type but are only rarely encountered. As a typical feature, they grow along and invade the lymphatics, with subcutaneous tumour cords being visible and/or palpable.^27^

In sarcoids, BPV1/2 infection predominantly involves dermal fibroblasts and is exclusively episomal in these cells.^30^, ^31^ Viral episomes replicate in synchrony with cell division thus assuring genome maintenance.^32^ This efficient form of PV amplification, and the ability of BPV1 and BPV2 to establish episomal infection also in apparently healthy skin of sarcoid‐affected horses^11^, ^33^ likely explain the frequently reported onset, progression, and reoccurrence of sarcoids following accidental or iatrogenic trauma.^5^ The role of trauma in the (re)activation of PV‐induced disease is widely recognised.^34^

Genetic analysis has mapped the transforming activities of BPV1 and BPV2 to the early genes E5, E6, and E7.^35^, ^36^, ^37^ The corresponding oncoproteins are expressed throughout tumour development and act in a combined manner. They assure the survival of infected host cells by inducing their immortalisation, hyperproliferation, and anoikis‐independent growth.^35^, ^36^, ^37^ The E6 oncoprotein acts as a transcriptional activator, abrogating the apoptotic and cell‐cycle arrest functions of the tumour suppressor p53 by downregulating CBP/p300 transcription. E6 also competitively binds to the focal adhesion protein paxillin, thus abrogating its interaction with other adhesion proteins and allowing infected cells to grow in an anchorage‐independent manner.^38^, ^39^, ^40^, ^41^ The E7 oncoprotein is co‐involved in this process via binding to p600.^40^, ^41^ The small hydrophobic E5 oncoprotein forms dimers and oligomers that interact with host cell regulatory proteins, most notably the PDGFβ‐receptor. Binding of E5 to this receptor leads to its sustained activation—a prerequisite for E5‐induced transformation of infected cells.^42^, ^43^, ^44^

## 2 IMMUNE RESPONSE TO BPV INFECTION IN EQUIDS

In the bovine host, BPV‐induced papillomas (“cow warts”) commonly regress within several months following the activation of an immune response that is mainly cellular. Anti‐BPV1/2 antibodies are only rarely detected in infected cattle. Immunohistochemical analysis of regressing BPV4‐associated papillomas has revealed the presence of high amounts of CD4^+^ T lymphocytes (T cells), but also γδ‐ and CD8^+^ T cells in the dermis underneath regressing lesions, whilst γδ and CD8^+^ T cells prevailed in the basal epidermal layer of these lesions.^3^, ^45^ More recently, Geisshüsler and colleagues provided evidence of high levels of CD8^+^ T cells also in equine sarcoids and adjacent tissue using immunofluorescence staining (IF). In addition, perilesional skin contained significantly higher amounts of CD4^+^ T cells than normal skin.^46^ This observation contrasts with the general opinion that the immune response to BPV infection and associated sarcoids in horses and other equids is poor.^47^

The host defence against pathogens relies on innate and adaptive immune mechanisms. As the first line of defence, the innate immune system acts immediately against an infectious intruder. Pattern recognition receptors (PRRs) such as Toll‐like receptors (TLRs) have a key role in this process. They are mainly expressed by immune cells and recognise conserved pathogen structures termed pathogen‐associated molecular patterns (PAMPs), as well as damage‐associated molecular patterns (DAMPs). Recognition of these patterns initiates an immune response that ultimately clears the microbial assault in about 90% of cases.^48^, ^49^ In mammals, the TLR family comprises 12 members that are grouped into several subfamilies according to the PAMPs/DAMPs they can recognise. TLR2, TLR4, and TLR9 are the most versatile TLRs in that they recognise structural patterns from bacteria, fungi, parasites and viruses. Interestingly, TLR4 can also identify host‐specific heat shock protein 60 and 70, as well as fibrinogen patterns. In addition, TLR4 is not only expressed by immune cells, but also by tumour cells.^50^ Little is known on the horse's innate immune response to BPV infection. However, there is evidence that TLR4 is downregulated in BPV1‐infected equine fibroblasts and primary sarcoid cells.^51^ Inhibition experiments conducted in BPV1 plasmid transfected equine fibroblasts demonstrated that TLR4 suppression is largely mediated by the viral proteins E2 and E7.^52^

T cells are also the major players in adaptive immunity. They activate B cell responses and, importantly, are also able to control intracellular pathogens such as viruses by inducing a cell‐mediated immune response and immune memory.^53^ To this aim, T cells recognise short antigen peptides presented on the cell surface by major histocompatibility complex class I or class II molecules (MHC I, MHC II). Whilst MHC II is mainly expressed by antigen‐presenting cells and commonly presents peptides stemming from pathogens circulating in the extracellular space, MHC I is generated by all nucleated cells and presents endogenous antigens such as viral proteins on the cell surface.^53^, ^54^ Intriguingly, there is ample evidence showing that HPV16 E5 and BPV1 E5 irreversibly inhibit the cell surface expression of MHC class I, thus compromising viral antigen presentation and allowing these viruses to escape immune surveillance.^55^, ^56^

To conclude, there are at least two mechanisms, that is, the downregulation of TLR4 by E2 and E7, and of MHC I by E5, that help explain the poor innate and adaptive immunogenicity of BPV infection and resulting sarcoid disease in equids.^47^

## 3 SPONTANEOUS REGRESSION OF EXPERIMENTAL PSEUDO‐SARCOIDS

In 1951, Olson and Cook were probably the first to establish a causal relationship between “cow wart viruses”, that is, BPV1/2, and sarcoids in horses. They purified infectious virions from bovine papillomas and applied the cell‐free preparation onto scarified skin of foals. The experiment resulted in the development of nodular skin lesions that were morphologically and histologically indistinguishable from sarcoids. However, in contrast to naturally acquired disease, the experimental lesions termed “pseudo‐sarcoids” resolved spontaneously.^57^ Analogous results were obtained by epidermal/intradermal inoculation of horses with BPV1/2 virions in the 1960s.^58^, ^59^ These early experiments were repeated in 2011 to establish an infection model for vaccination studies.^60^ Four yearlings that were confirmed to be BPV1/2 infection‐free were inoculated with cow wart‐derived infectious BPV1 virions on the left side of the neck (10 wheals with 5 × 10^7^ virions per wheal, and 10 wheals with 1 × 10^7^ virions per wheal). On the right side of the neck, the yearlings were injected intradermally with sarcoid‐derived fibroblasts containing episomal BPV1 DNA (six wheals with 2 × 10^5^ cells per wheal), virus‐free equine fibroblasts (six wheals with 2 × 10^5^ cells per wheal), BPV1 genome (five wheals with 360 ng BPV1 DNA per wheal) or phosphate buffered saline. The injection volume per wheal was uniformly 50 μL. Importantly, pseudo‐sarcoids developed in all four horses at cutaneous sites inoculated with BPV1 virions, whilst injection with other inocula had no apparent effect. Spontaneous tumour regression was complete in all horses after 36 weeks. Whilst inoculation with virions failed to induce a detectable amount of neutralising anti‐BPV1 antibodies, viral DNA (and mRNA) were detectable in peripheral blood mononuclear cells (PBMCs) from day seven post inoculation onwards. Overall, BPV1 DNA loads ranged between 0.2 and 7.5 copies per 10^3^ PBMCs, with loads consecutively increasing during tumour growth and then decreasing towards zero in the course of spontaneous tumour regression. No more viral DNA was detected in PBMCs after the lesions had resolved. PBMCs are immune cells, that is, T and B lymphocytes and NK cells, as well as monocytes, that is, macrophages and dendritic cells. Detection of BPV1 DNA and RNA in PBMCs hence indicates that spontaneous pseudo‐sarcoid regression was mediated by an effective cellular immune response induced by excess BPV1 antigen in the inoculum: As a matter of fact, each horse was intradermally injected with 6 × 10^8^ virions in total.^60^

## 4 SPONTANEOUS REGRESSION OF NATURALLY ACQUIRED SKIN LESIONS DIAGNOSED AS SARCOIDS

Transmission of BPV infection to equids is still an unresolved issue. Initially, BPV1/2 infection of equids was described as abortive, meaning that no infectious virions are produced in the non‐bovine host.^5^, ^30^, ^31^ Meanwhile, it has been demonstrated that BPV1/2 infection can be productive in equids, albeit at a low level.^33^, ^61^, ^62^, ^63^ Today, it is widely accepted that BPV1/2 transmission to equids is virion‐mediated and also occurs within equid populations^37^ possibly by direct contact, contaminated fomites, and/or flying insects feeding on sarcoids.^64^, ^65^, ^66^, ^67^ In each transmission scenario, a few infectious virions must suffice to produce infection. In an in‐vitro approach, equine primary fibroblasts were co‐cultured with decreasing amounts that is, 2 × 10^6^ to only 20 infectious BPV1 or BPV2 virions. Intriguingly, all virion doses produced stable infections, and at the fourth passage, average viral DNA loads had settled at a physiological level of around 150 copies per cell, irrespective of the number of virions used for in‐vitro infection.^68^ The minimal virion dose required for natural infection of animals or humans by PVs remains to be elucidated. In humans, there are indications that it is extremely low, possibly consisting of a single infectious HPV particle.^69^, ^70^, ^71^

Together with known PV‐mediated mechanisms of immune evasion, the suspected low number of virions required for successful infection, and the immortalisation of infected cells instead of their lysis may help explain why sarcoids can develop undetected by the immune system and thus persist.

This view is challenged by a few reports on the spontaneous regression of lesions diagnosed as equine sarcoids. In 1995, Broström presented retrospective data of 120 horses with clinically diagnosed sarcoids. In horses treated by surgical tumour excision (*n* = 64), the diagnosis was confirmed by histopathological examination of tumour sections. In 34 cases, horses were affected by “solitary lesions not requiring treatment” according to the veterinary practitioners, and the diagnosis of equine sarcoid was only based on clinical examination. Ten of these 34 untreated skin lesions spontaneously resolved within a time span of 0.5 to 7 years following veterinary consult.^72^

Berruex and colleagues studied the clinical course of sarcoids in a population of Franches‐Montagnes horses. Of 702 three‐year‐old animals, 83 were clinically diagnosed as being sarcoid‐affected based on the typical gross appearance and localisation of the skin lesions. Five to seven years later, 61 of the 83 horses were still available for clinical re‐examination. In 29 of the 61 horses, lesions had resolved without treatment and were classified as “cases of spontaneous regression”. These lesions had been clinically categorised as occult or verrucous sarcoids; one lesion was of small, nodular type.^29^ In another study, Cosandey and colleagues evaluated the potential of selected micro RNAs as diagnostic and prognostic sarcoid markers in Franches‐Montagnes and Swiss Warmblood horses. The study comprised a group of 19 individuals with spontaneously regressed lesions clinically diagnosed as sarcoids. The diagnosis was based on the presence of “typical morphologic features and distribution pattern” of the skin lesions. The self‐resolving lesions were generally described as “mild” sarcoids but not clinically classified.^73^

In 2010, Christen‐Clottu et al. reported on the efficacy of mistletoe extract (*Viscum album* extract; Iscador P, Weleda AG, Arlesheim, Switzerland) in the treatment of sarcoids. The study comprised 53 horses bearing a total of 444 clinically diagnosed sarcoids. In 42 horses, the diagnosis was partly confirmed by histopathological examination of excised lesions or biopsies of selected tumours, excluding lesions clinically classified as occult sarcoids. In 11 horses, sarcoid diagnosis was solely based on clinical examination irrespective of the type of lesions. The authors also state that on tumour level, clinical sarcoid diagnosis was not confirmed by histopathological examination in the “majority” of the cases.^74^

In the control group receiving placebo (i.e., subcutaneous injections with physiological saline solution), regression was observed in 14.3% of horses bearing 13.2% of sarcoids, that is, individuals affected by one or two lesions of mild verrucous or presumed occult type (clinical diagnosis only). There is no information on how many diagnoses of verrucous sarcoid were confirmed histopathologically.^74^

Although interesting, the above‐mentioned studies have a common weakness: sarcoid diagnosis was made by clinical examination and not confirmed by histopathological analysis of tumour tissue in the vast majority of cases. In addition, no attempts were made to screen superficial tumour scrapings or swabs by BPV1/2 PCR, although this method constitutes a rapid and reliable, non‐invasive method to validate clinical diagnosis of sarcoid.^5^, ^23^, ^36^, ^75^ As a consequence, it is impossible to tell how many of the reported spontaneously regressing skin lesions truly corresponded to sarcoids.

## 5 CONCLUSION

In an experimental setting, pseudo‐sarcoids induced by epidermal or intradermal application of excess BPV1/2 virions regress spontaneously within several weeks or months.^6^, ^57^, ^58^, ^59^, ^60^ This phenomenon is likely based on a robust cellular immune response.^60^ Under natural conditions, equids are exposed to far lower numbers of infectious BPV particles, so that infection can probably be prevented or cleared in many cases.^3^, ^54^ Sometimes, however, infection is successfully established and usually persists thanks to viral immune evasion and genome maintenance mechanisms, leading to sarcoid disease.^5^, ^47^ A number of single lesions of mild occult or mild verrucous type may not adhere to this general rule. They may resolve spontaneously after several months, as similarly observed for BPV1/2‐induced cow warts.^8^

So far available data suggest that spontaneous regression of sarcoids constitutes an exceptional event that is confined to mild lesions of occult and possibly verrucous type.

## FUNDING INFORMATION

This work received no funding.

## CONFLICT OF INTEREST STATEMENT

The author has declared no conflicting interests.

## AUTHOR CONTRIBUTIONS

**Sabine Brandt:** Conceptualization; writing – original draft; validation; writing – review and editing.

## DATA INTEGRITY STATEMENT

No new data were created or analysed in this study.

## Data Availability

Data sharing is not applicable to this article as no new data were created or analysed in this study.

## References

[1] Campo MS . Introduction. In: Campo MS , editor. Papillomavirus research: from natural history to vaccines and beyond. 1st ed. Norfolk, UK: Caister Academic Press; 2006. p. 1–2.

[2] Doorbar J . The papillomavirus life cycle. J Clin Virol. 2005;32(Suppl 1):S7–S15. 15753007 10.1016/j.jcv.2004.12.006

[3] Campo MS . Bovine papillomavirus: old system, new lessons? In: Campo MS , editor. Papillomavirus research: from natural history to vaccines and beyond. 1st ed. Norfolk, UK: Caister Academic Press; 2006. p. 373–387.

[4] Doorbar J , Quint W , Banks L , Bravo IG , Stoler M , Broker TR , et al. The biology and life‐cycle of human papillomaviruses. Vaccine. 2012;30(Suppl 5):F55–F70. 23199966 10.1016/j.vaccine.2012.06.083

[5] Chambers G , Ellsmore VA , O'Brien PM , Reid SW , Love S , Campo MS , et al. Association of bovine papillomavirus with the equine sarcoid. J Gen Virol. 2003;84:1055–1062. 12692268 10.1099/vir.0.18947-0

[6] Hainisch EK , Abel‐Reichwald H , Shafti‐Keramat S , Pratscher B , Corteggio A , Borzacchiello G , et al. Potential of a BPV1 L1 VLP vaccine to prevent BPV1‐ or BPV2‐induced pseudo‐sarcoid formation and safety and immunogenicity of EcPV2 L1 VLPs in the horse. J Gen Virol. 2016;98(2):230–241. 10.1099/jgv.0.000673 28284277

[7] Shafti‐Keramat S , Schellenbacher C , Handisurya A , Christensen N , Reininger B , Brandt S , et al. Bovine papillomavirus type 1 (BPV1) and BPV2 are closely related serotypes. Virology. 2009;393:1–6. 19729180 10.1016/j.virol.2009.07.036 PMC3792341

[8] Borzacchiello G , Roperto F . Bovine papillomaviruses, papillomas and cancer in cattle. Vet Res. 2008;39:45. 18479666 10.1051/vetres:2008022

[9] Lunardi M , de Alcantara BK , Otonel RA , Rodrigues WB , Alfieri AF , Alfieri AA . Bovine papillomavirus type 13 DNA in equine sarcoids. J Clin Microbiol. 2013;51:2167–2171. 23637294 10.1128/JCM.00371-13 PMC3697707

[10] Cutarelli A , Buonavoglia A , Fusco G , Pellicano R , Napoletano M , Brandt S , et al. Accurate identification of bovine deltapapillomavirus in equine sarcoids by ddPCR. Sci Rep. 2025;15:29414. 40790360 10.1038/s41598-025-15353-6 PMC12340048

[11] Carr EA , Theon AP , Madewell BR , Griffey SM , Hitchcock ME . Bovine papillomavirus DNA in neoplastic and nonneoplastic tissues obtained from horses with and without sarcoids in the western United States. Am J Vet Res. 2001;62:741–744. 11341396 10.2460/ajvr.2001.62.741

[12] Wobeser BK , Davies JL , Hill JE , Jackson ML , Kidney BA , Mayer MN , et al. Epidemiology of equine sarcoids in horses in western Canada. Can Vet J. 2010;51:1103–1108. 21197201 PMC2942047

[13] Bogaert L , Martens A , Depoorter P , Gasthuys F . Equine sarcoids ‐ Part 1: clinical presentation and epidemiology. Vlaams Diergeneeskd Tijdschr. 2008;77:2–9.

[14] Scott DW , Miller WH Jr . Sarcoid. In: Scott DW , Miller WH Jr , editors. Equine Dermatology. St. Louis: Saunders Elsevier; 2003. p. 719–731.

[15] Ireland JL , Wylie CE , Collins SN , Verheyen KL , Newton JR . Preventive health care and owner‐reported disease prevalence of horses and ponies in Great Britain. Res Vet Sci. 2013;95:418–424. 23768693 10.1016/j.rvsc.2013.05.007

[16] Reid SWJ , Gettinby G , Fowler JN , Ikin P . Epidemiologic observations on sarcoids in a population of donkeys (Equus‐Asinus). Vet Rec. 1994;134:207–211. 8171807 10.1136/vr.134.9.207

[17] Mele M , Gerber V , Straub R , Gaillard C , Jallon L , Burger D . Prevalence of hereditary diseases in three‐year‐old horses of the Freiberger breed. Schweiz Arch Tierheilkd. 2007;149:151–159. 17461390 10.1024/0036-7281.149.4.151

[18] Studer S , Gerber V , Straub R , Brehm W , Gaillard C , Luth A , et al. Prevalence of hereditary diseases in three‐year‐old Swiss Warmblood horses. Schweiz Arch Tierheilkd. 2007;149:161–171. 17461391 10.1024/0036-7281.149.4.161

[19] Joch D . Sarkoiderkrankung in Bezug auf Alter, Rasse und Geschlecht. Master thesis, University of Veterinary Medicine, Vienna, Austria. 2018.

[20] Mohammed HO , Rebhun WC , Antczak DF . Factors associated with the risk of developing sarcoid tumours in horses. Equine Vet J. 1992;24:165–168. 1606927 10.1111/j.2042-3306.1992.tb02808.x

[21] Valentine BA . Survey of equine cutaneous neoplasia in the Pacific Northwest. J Vet Diagn Invest. 2006;18:123–126. 16566271 10.1177/104063870601800121

[22] Nasir L , Reid SWJ . Bovine papillomaviruses and equine sarcoids. In: Campo MS , editor. Papillomavirus research: from natural history to vaccines and beyond. 1st ed. Norfolk, UK: Caister Academic Press; 2006. p. 389–397.

[23] Hainisch EK , Brandt S . Equine sarcoids. In: Robinson NE , Sprayberry KA , editors. Robinson's current therapy in equine medicine. 7th ed. St. Louis: Saunders Elsevier; 2015 Chapter 99.

[24] Haspeslagh M , Vlaminck LE , Martens AM . Treatment of sarcoids in equids: 230 cases (2008‐2013). J Am Vet Med Assoc. 2016;249:311–318. 27439349 10.2460/javma.249.3.311

[25] Knottenbelt DC . Sarcoid. In: Knottenbelt DC , editor. Pascoe's principles and practice of equine dermatology. London, UK: Saunders Elsevier; 2009. p. 387–407.

[26] Knottenbelt DC . The equine sarcoid: why are there so many treatment options? Vet Clin North Am Equine Pract. 2019;35:243–262. 31097356 10.1016/j.cveq.2019.03.006

[27] Knottenbelt DC . A suggested clinical classification for the equine sarcoid. Clin Tech Equine Pract. 2005;4:278–295.

[28] Knottenbelt DC , Kelly DF . The diagnosis and treatment of periorbital sarcoid in the horse: 445 cases from 1974 to 1999. Vet Ophthalmol. 2000;3:169–191. 11397301 10.1046/j.1463-5224.2000.00119.x

[29] Berruex F , Gerber V , Wohlfender FD , Burger D , Koch C . Clinical course of sarcoids in 61 Franches‐Montagnes horses over a 5‐7 year period. Vet Q. 2016;36:189–196. 27327513 10.1080/01652176.2016.1204483

[30] Amtmann E , Muller H , Sauer G . Equine connective tissue tumors contain unintegrated bovine papilloma virus DNA. J Virol. 1980;35:962–964. 6252350 10.1128/jvi.35.3.962-964.1980 PMC288890

[31] Lancaster WD . Apparent lack of integration of bovine papillomavirus DNA in virus‐induced equine and bovine tumor cells and virus‐transformed mouse cells. Virology. 1981;108:251–255. 6258289 10.1016/0042-6822(81)90433-5

[32] Angelos JA , Marti E , Lazary S , Carmichael LE . Characterization of BPV‐like DNA in equine sarcoids. Arch Virol. 1991;119:95–109. 1650553 10.1007/BF01314326

[33] Brandt S , Haralambus R , Shafti‐Keramat S , Steinborn R , Stanek C , Kirnbauer R . A subset of equine sarcoids harbours BPV‐1 DNA in a complex with L1 major capsid protein. Virology. 2008;375:433–441. 18395238 10.1016/j.virol.2008.02.014

[34] Chow LT , Broker TR . Mechanisms and regulation of papillomavirus DNA replication. In: Campo MS , editor. Papillomavirus research: from natural history to vaccines and beyond. 1st ed. Norfolk, UK: Caister Academic Press; 2006. p. 53–71.

[35] Howley PM , Lowy DR . Papillomaviruses and their replication. In: Knipe DM , Howley PM , editors. Fields virology. 4th ed. Philadelphia, PA, USA: Lippincott Williams & Wilkins; 2001. p. 2197–2230.

[36] Nasir L , Brandt S . Papillomavirus associated diseases of the horse. Vet Microbiol. 2013;167:159–167. 24016387 10.1016/j.vetmic.2013.08.003

[37] Nasir L , Campo MS . Bovine papillomaviruses: their role in the aetiology of cutaneous tumours of bovids and equids. Vet Dermatol. 2008;19:243–254. 18927950 10.1111/j.1365-3164.2008.00683.x

[38] Tong X , Howley PM . The bovine papillomavirus E6 oncoprotein interacts with paxillin and disrupts the actin cytoskeleton. Proc Natl Acad Sci U S A. 1997;94:4412–4417. 9114003 10.1073/pnas.94.9.4412 PMC20736

[39] Yuan Z , Gault EA , Campo MS , Nasir L . Different contribution of bovine papillomavirus type 1 oncoproteins to the transformation of equine fibroblasts. J Gen Virol. 2011;92:773–783. 21177927 10.1099/vir.0.028191-0

[40] DeMasi J , Huh KW , Nakatani Y , Munger K , Howley PM . Bovine papillomavirus E7 transformation function correlates with cellular p600 protein binding. Proc Natl Acad Sci U S A. 2005;102:11486–11491. 16081543 10.1073/pnas.0505322102 PMC1182553

[41] Wise‐Draper TM , Wells SI . Papillomavirus E6 and E7 proteins and their cellular targets. Front Biosci. 2008;13:1003–1017. 17981607 10.2741/2739

[42] Borzacchiello G . Bovine papillomavirus on the scene of crime: is E5 oncogene the only guilty party? Infect Agent Cancer. 2013;8:26. 23829702 10.1186/1750-9378-8-26 PMC3704702

[43] Borzacchiello G , Roperto F , Campo MS , Venuti A . 1st international workshop on papillomavirus E5 oncogene‐a report. Virology. 2010;408:135–137. 20947117 10.1016/j.virol.2010.09.015

[44] Suprynowicz FA , Campo MS , Schlegel R . Biologic activities of papillomavirus E5 proteins. In: Campo MS , editor. Papillomavirus research: from natural history to vaccines and beyond. 1st ed. Norfolk, UK: Caister Academic Press; 2006. p. 97–114.

[45] Knowles G , Grindlay GJ , Campo MS , Chandrachud LM , O'Neil BW . Linear B‐cell epitopes in the N‐terminus of L2 of bovine papillomavirus type 4. Res Vet Sci. 1997;62:289–291. 9300551 10.1016/s0034-5288(97)90207-1

[46] Geisshusler H , Marti E , Stoffel MH , Kuhni K , Stojiljkovic A , von Tscharner C , et al. Quantitative analysis of infiltrating immune cells and bovine papillomavirus type 1 E2‐positive cells in equine sarcoids. Vet J. 2016;216:45–52. 27687925 10.1016/j.tvjl.2016.06.016

[47] Brandt S . Immune response to bovine papillomavirus type 1 in equine sarcoid. Vet J. 2016;216:107–108. 27687935 10.1016/j.tvjl.2016.07.012

[48] Kumar H , Kawai T , Akira S . Pathogen recognition by the innate immune system. Int Rev Immunol. 2011;30:16–34. 21235323 10.3109/08830185.2010.529976

[49] Stanley MA . Immunobiology of papillomavirus infections. J Reprod Immunol. 2001;52:45–59. 11600177 10.1016/s0165-0378(01)00113-9

[50] Huang B , Zhao J , Li H , He KL , Chen Y , Chen SH , et al. Toll‐like receptors on tumor cells facilitate evasion of immune surveillance. Cancer Res. 2005;65:5009–5014. 15958541 10.1158/0008-5472.CAN-05-0784

[51] Yuan ZQ , Nicolson L , Marchetti B , Gault EA , Campo MS , Nasir L . Transcriptional changes induced by bovine papillomavirus type 1 in equine fibroblasts. J Virol. 2008;82:6481–6491. 18434409 10.1128/JVI.00429-08 PMC2447052

[52] Yuan ZQ , Bennett L , Campo MS , Nasir L . Bovine papillomavirus type 1 E2 and E7 proteins down‐regulate Toll Like Receptor 4 (TLR4) expression in equine fibroblasts. Virus Res. 2010;149:124–127. 20109504 10.1016/j.virusres.2010.01.008

[53] Janeway CAJ , Travers P , Walport M , Shlomchik MJ . The recognition and effector mechanisms of adaptive immunity. Immunobiology: the immune system in health and disease. 6th ed. New York: Garland Science Publishing; 2005. p. 24–36.

[54] Stanley M . Immunobiology of papillomaviruses. In: Campo MS , editor. Papillomavirus research: from natural history to vaccines and beyond. 1st ed. Norfolk, UK: Caister Academic Press; 2006. p. 311–319.

[55] Marchetti B , Gault EA , Cortese MS , Yuan Z , Ellis SA , Nasir L , et al. Bovine papillomavirus type 1 oncoprotein E5 inhibits equine MHC class I and interacts with equine MHC I heavy chain. J Gen Virol. 2009;90:2865–2870. 19675187 10.1099/vir.0.014746-0

[56] Ashrafi GH , Brown DR , Fife KH , Campo MS . Down‐regulation of MHC class I is a property common to papillomavirus E5 proteins. Virus Res. 2006;120:208–211. 16780984 10.1016/j.virusres.2006.02.005

[57] Olson C Jr , Cook RH . Cutaneous sarcoma‐like lesions of the horse caused by the agent of bovine papilloma. Proc Soc Exp Biol Med. 1951;77:281–284. 14854020 10.3181/00379727-77-18750

[58] Ragland WL , Spencer GR . Attempts to relate bovine papilloma virus to the cause of equine sarcoid: equidae inoculated intradermally with bovine papilloma virus. Am J Vet Res. 1969;30:743–752. 5813668

[59] Voss JL . Transmission of equine sarcoid. Am J Vet Res. 1969;30:183–191. 5392976

[60] Hartl B , Hainisch EK , Shafti‐Keramat S , Kirnbauer R , Corteggio A , Borzacchiello G , et al. Inoculation of young horses with bovine papillomavirus type 1 virions leads to early infection of PBMCs prior to pseudo‐sarcoid formation. J Gen Virol. 2011;92:2437–2445. 21715602 10.1099/vir.0.033670-0 PMC5034893

[61] Bogaert L , Martens A , Kast WM , Van Marck E , De Cock H . Bovine papillomavirus DNA can be detected in keratinocytes of equine sarcoid tumors. Vet Microbiol. 2010;146:269–275. 21095508 10.1016/j.vetmic.2010.05.032

[62] Brandt S , Tober R , Corteggio A , Burger S , Sabitzer S , Walter I , et al. BPV‐1 infection is not confined to the dermis but also involves the epidermis of equine sarcoids. Vet Microbiol. 2011;150:35–40. 21242040 10.1016/j.vetmic.2010.12.021

[63] Wilson AD , Armstrong EL , Gofton RG , Mason J , De Toit N , Day MJ . Characterisation of early and late bovine papillomavirus protein expression in equine sarcoids. Vet Microbiol. 2013;162:369–380. 23123175 10.1016/j.vetmic.2012.10.010

[64] Abel‐Reichwald H , Hainisch EK , Zahalka S , Corteggio A , Borzacchiello G , Massa B , et al. Epidemiologic analysis of a sarcoid outbreak involving 12 of 111 donkeys in Northern Italy. Vet Microbiol. 2016;196:85–92. 27939161 10.1016/j.vetmic.2016.10.021

[65] Finlay M , Yuan Z , Burden F , Trawford A , Morgan IM , Campo MS , et al. The detection of bovine papillomavirus type 1 DNA in flies. Virus Res. 2009;144:315–317. 19409942 10.1016/j.virusres.2009.04.015

[66] Kemp‐Symonds JG . The detection and sequencing of bovine papillomavirus type 1 and 2 DNA from Musca autumnalis (Diptera: Muscidae) face flies infesting sarcoid‐affected horses. MSc thesis, Royal Veterinary College, London, UK. 2000.

[67] Bogaert L , Martens A , De Baere C , Gasthuys F . Detection of bovine papillomavirus DNA on the normal skin and in the habitual surroundings of horses with and without equine sarcoids. Res Vet Sci. 2005;79:253–258. 16054896 10.1016/j.rvsc.2004.12.003

[68] Hainisch EK , Jindra C , Reicher P , Miglinci L , Brodesser DM , Brandt S . Bovine papillomavirus type 1 or 2 Virion‐infected primary fibroblasts constitute a near‐natural equine sarcoid model. Viruses. 2022;14(12):2658. 10.3390/v14122658 36560661 PMC9781842

[69] Galloway DA . Human papillomaviruses: a growing field. Genes Dev. 2009;23:138–142. 19171777 10.1101/gad.1765809 PMC2763491

[70] Giroglou T , Florin L , Schafer F , Streeck RE , Sapp M . Human papillomavirus infection requires cell surface heparan sulfate. J Virol. 2001;75:1565–1570. 11152531 10.1128/JVI.75.3.1565-1570.2001 PMC114064

[71] Patterson NA , Smith JL , Ozbun MA . Human papillomavirus type 31b infection of human keratinocytes does not require heparan sulfate. J Virol. 2005;79:6838–6847. 15890923 10.1128/JVI.79.11.6838-6847.2005 PMC1112118

[72] Brostrom H . Equine sarcoids. A clinical and epidemiological study in relation to equine leucocyte antigens (ELA). Acta Vet Scand. 1995;36:223–236. 7484549 10.1186/BF03547691 PMC8095413

[73] Cosandey J , Hamza E , Gerber V , Ramseyer A , Leeb T , Jagannathan V , et al. Diagnostic and prognostic potential of eight whole blood microRNAs for equine sarcoid disease. PLoS One. 2021;16:e0261076. 34941894 10.1371/journal.pone.0261076 PMC8699634

[74] Christen‐Clottu O , Klocke P , Burger D , Straub R , Gerber V . Treatment of clinically diagnosed equine sarcoid with a mistletoe extract (Viscum album austriacus). J Vet Intern Med. 2010;24:1483–1489. 21039860 10.1111/j.1939-1676.2010.0597.x

[75] Haralambus R , Burgstaller J , Klukowska‐Rotzler J , Steinborn R , Buchinger S , Gerber V , et al. Intralesional bovine papillomavirus DNA loads reflect severity of equine sarcoid disease. Equine Vet J. 2010;42:327–331. 20525051 10.1111/j.2042-3306.2010.00078.x

